# Effects of Parental Internalizing and Externalizing Behavior Problems on Children’s Limbic Brain Structures—An MRI Study

**DOI:** 10.3390/brainsci12101319

**Published:** 2022-09-29

**Authors:** Zainab Albar, Abdus Sattar

**Affiliations:** Department of Population and Quantitative Health Sciences, School of Medicine, Case Western Reserve University, Cleveland, OH 44106, USA

**Keywords:** parenting, behavioral measures, child development, brain imaging, quantile regression

## Abstract

Parental behavior problems have long-term effects on children’s limbic brain structures and functions. Parental behavior problems-related brain changes in children may lead to mental disorders and behavior dysfunction later in life. However, our understanding of the relationship between parental behavior and children’s brain structures is less obvious when children and adolescents are studied in a general population without mental disorders. The majority of studies on the relationship between parental behavior and adolescent brain structure have been focused on severe forms of the following parental behavior problems: (1) internalizing behavior associated with mood and anxiety disorders, and (2) externalizing behavior associated with substance use and violence. A few studies examined the effect of normative variations or subtle differences in parental behavior. Therefore, we utilized a large study—Adolescent Brain Cognitive Development (ABCD)—to determine relationships between normative variation in parental internalizing and externalizing behavior and limbic brain structures in children and adolescents without mental disorders. Quantile (median) regression models were used to compute associations between parental behavior and children’s limbic structures. We found that parental internalizing and externalizing behaviors are uniquely associated with children’s limbic structures after adjustment for biological confounders and parental socioeconomic status. Our findings indicate that normative parental behavior may have a significant early influence on limbic structures of normally developing children and adolescents. Accelerated or delayed limbic structure maturation may account for children’s and adolescents’ behavioral inadequacies and a risk of developing specific mood disorders or substance abuse problems later in life.

## 1. Introduction

Parental behavior problems are a common plight that impacts children’s behavior, cognitive function, and emotional development [1,2]. Parental behavior problems also have lasting effects on brain structures and functions [3,4]. Several studies suggest that changes in structural and functional brain development caused by parental behavior problems may contribute to mental disorders and behavior dysfunction later in life [5]. Despite these documented findings, the association between parental behavior and brain structures and mental disorders is less obvious when children or adolescents are studied in a general population without any mental disorders.

Parental behavior problems can be classified into two distinct categories: (1) an internalizing dimension that indicates a tendency for mood and anxiety disorders such as depression, bipolar disorder, and generalized anxiety disorder [6]; and (2) an externalizing dimension that indicates a tendency for antisocial behavior and substance abuse disorders [6]. In prior studies, parents’ depression (an internalized disorder) was associated with a smaller putamen (a subcortical brain structure) in their children [7], and maternal anxiety was associated with changes in error-related negativity (i.e., anterior cingulate cortex activity) in their children, as well as an increased risk of developing anxiety during adolescence [8]. Nevertheless, it has been reported that harsh or aggressive parental behavior (externalized disorder) affects prefrontal cortex, anterior cingulate, and amygdala development [9], and aggressive maternal behavior was associated with increased thickness of the right superior frontal gyrus, greater area of the lateral parietal lobe, and smaller volume of Nucleus Accumbens in males [10].

Most of the studies on the relationship between parental behavior problems and altered brain structure and function were limited by a variety of reasons. For example, many studies were conducted retrospectively by examining the association between childhood adversities and adulthood mental disorders. These studies recruited subjects primarily in adulthood and a few in adolescence, with varying ages and small sample sizes, and used retrospective self-reported data about parental behavior during their childhood period [5,11,12]. Furthermore, the majority of studies on the relationship between parental behavior and adolescent brain structure have been focused on severe forms of parental behavior problems associated with mental illnesses, substance use, violence, or criminality [13]. 

In general, severe forms of parental behavior problems were associated with functional irritability in children’s limbic structures and delayed development of the hippocampus, orbitofrontal, and anterior cingulate cortex [11,14,15]. All these brain structural and functional changes were related to poor functional and mental health outcomes later in life [10,11,13]. Therefore, it is important to determine a relationship between normative variation in parental behavior (in absence of child maltreatment) and brain structure in children’s brains in order to provide probable explanations for mental health disorder and behavioral dysfunction that may occur during late childhood or adolescence [16].

A few studies examined the effects of normative variations or subtle differences in parental behavior that may be considered less intense and have a milder effect on the brains and mental health of children and adolescents [4]. Our understanding of how brain structure and function varies during childhood and adolescence in relation to a normative range of parental behavior remains an open question. Thus, the goal of this study was to examine the relationships between normative variation in parental behavior and limbic brain structures in children and adolescents without mental health disorders by using a large and representative children’s and adolescents’ population from the United States. A deeper understanding of the relationship between normative parenting and brain development is also important for identifying targets for parenting interventions to reduce adolescents’ risk of mental health disorders and behavioral dysfunction [13]. 

Currently, the neuroimaging studies [4,5] have focused on limbic brain structures in order to assess the association between various forms of parental behavior and developmental changes in children’s brains. The limbic structures are key regions of interest because they are involved in emotion and behavior regulation, as well as maintaining mental health and assisting the body in responding to stress [17]. The cortical parts of limbic structures are the orbitofrontal cortex (OFC) and cingulate cortex, which have been related to cognitive control of emotions and behaviors [18]. The OFC and cingulate cortex develop slowly, with steady reductions in cortical grey matter volume, thickness, and surface area from adolescence to the third decade of life [19].

The subcortical parts of limbic structures are the amygdala and hippocampus; they have been implicated in the perception and storage of emotion stimuli, and in transmitting information about emotional content for further processing [20]. The hippocampus and amygdala have a curvilinear growth pattern, with increasing volume during adolescence, and are sexually dimorphic structures that begin to reduce in volume by 20 years of age for males and 13 years for females [21].

For our analysis, we used data from the Adolescent Brain Cognitive Development (ABCD) study (release 3.0). It is the largest study of brain development and child well-being to date [22]. The ABCD Study collected data on stress, neurodevelopment, and psychiatric symptoms in 11,878 children. The study is unique in that it focused on children ages eight to eleven, when their limbic structures, gray matter brain growth, and white matter tract expansion are most sensitive [23]. Given the rapid brain reorganization during late childhood and early adolescence, parental behaviors are particularly likely to impact the structure of the brain during this time [24]. 

In summary, the goal of this study is to examine the relationships between parental internalizing and externalizing behaviors and limbic brain structures as the regions of interest (ROI) in children and adolescents drawn from a general population without mental health disorders. We will apply a robust analysis method, quantile regression, in the entire distribution of an ROI for a global understanding of the association. While a mean or median regression focuses association between the center of an ROI and the exposure variable, quantile regression examines association simultaneously between the entire distribution of an ROI and the exposure of interest. We will examine the volume, thickness, and surface area of the orbitofrontal cortex, cingulate cortex, and its subregions (rostral and caudal anterior cingulate, isthmus cingulate, and posterior cingulate), volume of hippocampus, and amygdala. We will further stratify our analysis by gender to account for differences in brain development between the genders [25].

## 2. Materials and Methods

The ABCD Study is the largest ongoing study on brain development and child health in the United States, representing the sociodemographic diversity in 21 sites across the U.S. [22]. The study gathered baseline information on multimodal brain imaging; extensive cognitive, psychiatric, and behavioral assessments; and family environment in 11,878 typically developed children ages eight to eleven years old. The narrow age range (8 to 11 years) enabled us to examine a wide range of relationships during a sensitive developmental period, relationships between parental behavior and children’s limbic structures at a stage when many mental disorders could arise [24]. The ABCD Study recruited children using a stratified probability sampling method to reduce the risk of any known systematic bias and maintained heterogeneity between children to increase representativeness of the study population. Details of the study design and rationale, as well as the process for recruiting participants and getting their approval consent, can be found in studies by Barch et al. [26] and Garavan et al. [22]. 

Because we are examining the relationship between parental behavior and children’s brain ROI, parental behavior is the study exposure variable or independent variable. Parental behavior was assessed using two broad-spectrum scales taken from the Achenbach Adult Self-Report (ASR) instrument: internalizing (r = 0.89, α = 0.93), and externalizing (r = 0.91, α = 0.89) behavior problem scales [27]. This ASR instrument has 120 problem items distributed among eight syndrome scales that are used to evaluate an adult’s behavioral, emotional, mental, and social interactions over the preceding six months. Each item is rated on a scale of 0 to 2, with “0” indicating that it is not true, “1” indicating that it is somewhat true, and “2” indicating that it is definitely true. When the score on problem items is added together, a raw score ranging from 0 to 240 is generated, as well as t-scores in respect to norms for each gender at the ages of 18–59, based on national probability samples. The broad-spectrum scale for internalizing behavior problems is formed by combining the Anxious/Depressed (18-item), Withdrawn (9-item), and Somatic Complaints (12-item) syndrome scales. The broad-spectrum scale for externalizing behavior problems is formed by combining the syndrome scales Aggressive Behavior (15 items), Rule-Breaking Behavior (14 items), and Intrusive Behavior (6 items). The t-score interpretation for parent internalizing and externalizing behavior is as follows: a score of 64 or less is considered normal behavior, 65 to 69 is considered borderline clinical, and 70 or more is considered clinical range of psychopathology symptoms and behavioral dysfunction. The majority of parent t-scores in the ABCD study are below 65 (n = 11,015 or 92.73 %); as such we intend to use the term “parental behavior” for the study’s exposure rather than symptoms, which are synonymous with “psychopathology symptoms”.

Our outcome variables are children’s brain ROI that include the OFC, cingulate cortex, hippocampus, and amygdala. The ABCD Data Analysis and Informatics Center (DAIC) performs centralized processing and analysis of MRI data for all imaging modality. The DAIC described the acquisition of MRI images, correction for distortions and head motion, cross modality registration, and manual quality control performed prior to full image processing in a study by Hagler et al. [28]. 

For sMRI-specific preprocessing, T1w and T2w structural images are corrected for gradient nonlinearity distortions using scanner-specific, nonlinear transformations provided by MRI scanner manufacturers. Then, intensity inhomogeneity correction is performed by applying smoothly varying, estimated B1-bias fields, using sparse spatial smoothing and white matter segmentation. Lastly, images are rigidly registered and resampled into alignment with an averaged reference brain (roughly at the anterior commissure/posterior commissure) in standard space, facilitating standardized viewing and analysis of brain structure [28]. 

The cortical surface reconstruction and subcortical segmentation are carried out using Free Surfer v5.3, which offers methods for quantifying several morphometric measures of the brain (e.g., cortical volume, thickness, and surface area). Cortical gray matter voxels (OFC and cingulate cortex) are labeled based on surface-based nonlinear registration to the Desikan-Killiany atlas [29]. Subcortical structures (amygdala and hippocampus) are labeled using an automated volumetric segmentation process based on the Fischl atlas [30]. 

We performed statistical analysis of ABCD Study data to understand changes in children’s brain ROI volumes in relation to parental internalizing and externalizing behaviors. We studied quality of the data by running frequency analyses, cross-tabulation, and summary statistics for continuous and categorical variables, as appropriate. We examined outliers in outcome variables (children’s brain ROI) using box plots and quantile–quantile (Q-Q) plots. Plausibility of the outliers was assessed from both practical, anatomical, and biological considerations. There are missing values in some of the covariates (e.g., income) and outcome variables (brain ROI). However, the percentage of missing is small (less than 10%) and missing values in the outcome variables are not related to the exposure of interest (parental internalizing and externalizing behaviors). Thus, we assume that the missing mechanism is missing completely at random in our further statistical analyses. We assessed normality of the outcome data distributions by generating Q-Q plots, as well as skewness and kurtosis tests. 

The outcome variables are not normally distributed (see Figure S1 in Supplemental Materials), and no existing transformations (including Box–Cox transformation) can make the distribution normal. Therefore, we conducted further statistical analysis using nonparametric approaches such as Spearman correlations and quantile (median) regression. 

Associations between parental internalizing and externalizing behaviors and children’s brain ROI are studied using Spearman correlations. Unadjusted and adjusted multivariable quantile regression models with robust standard error (SE) method are used to study the relationship between exposure and outcomes (brain ROI). Adjusted multivariable model included child’s age in years, binary race (white vs. nonwhite), gender (male vs. female), and parental marital status (married, single or other), parental education (1 “0-13G/GED”, 2 “College/Associate degree”, 3 “Bachelor”, 4 “Postgraduate education”) and yearly total income (1 “<$5k” to 10 “>$200k”) as the potential confounders or covariates. After the model fitting, we created quantile regression plots to investigate the association between exposure and outcomes across the entire range of brain ROI volume quantiles. The analysis was then stratified based on gender and subregions of the cingulate cortex. Note that for this exploratory study of the selected brain ROI, the *p*-value correction methodology was not applied. Instead, we applied the American statistical association’s guideline on the *p*-value [31]. In accordance with ASA guidelines, we estimated the associations of parental internalizing (and externalizing) variables in terms of regression coefficients β and its 95% confidence intervals. We accessed the ABCD study data on 10 February 2021, and performed all statistical analyses using StataCorp. 2019. Stata Statistical Software: Release 16. College Station, TX, USA: StataCorp LLC and R Core Team (2021). R: A language and environment for statistical computing. R Foundation for Statistical Computing, Vienna, Austria. URL https://www.R-project.org/ (accessed on 10 February 2021) R version 4.1.2. 

## 3. Results

Demographic characteristics of the ABCD Study participants are presented in Table 1. This study consists of 11,878 children, ages between 8 to 11 years, with 5682 females (47.80%). Participants of this study were 64.30% White, 16.00% African American, and 19.80% other races. About one fifth of the children are Hispanic (19.81%). The majority of the children live with their married parents or couples (73.70%) and most of the parents have at least a bachelor’s degree (53.90%). Almost a third of the families have a yearly income of less than $50,000.

Since parental internalizing or externalizing behavior are the exposure variables of this study, we presented these variables (in terms of t-score) summary statistics in a supplemental table, Table S1. Most of the parents’ (92.73% for internalizing and 95.49% for externalizing behavior) t-scores fall within normal range. The parental internalizing t-score variable is negatively associated with both hemispheres of children’s cingulate cortex, hippocampi, and amygdala (*p*-value ≤ 0.05), while the externalizing t-score is negatively associated with both hemispheres’ cingulate cortex surface area and hippocampi volumes (see Figures S2 and S3 in Supplemental Materials). 

We present the association among parental internalizing and externalizing variables and children’s brain ROI in Table 2 and Table 3, respectively. Using the median regression, we found children with higher parental internalizing behavior scores (Table 2) have an increased right hemisphere (RH) cingulate cortex volume by 3.75 mm^3^ (95% CI: [0.13 to 7.37 mm^3^], *p* = 0.042), and reduced hippocampi and amygdala volume in both hemispheres by −0.54 to −1.01 mm^3^ with *p*-value ≤ 0.05. We also found that children with higher parental externalizing behavior scores (Table 3) have a reduced left hemisphere (LH) OFC by −4.89 mm^3^ (95% CI: −8.48 to −1.31 mm^3^], *p* ≤ 0.01), LH cingulate cortex volume by −5.99 mm^3^ (95% CI: [−10.04 to −1.93 mm^3^], *p* ≤ 0.01) and reduced hippocampi by −1.4 mm^3^ with *p*-value ≤ 0.01 in both hemispheres. Children with higher parental externalizing behavior also have reduced LH orbitofrontal and cingulate cortex surface area, while none of the ROI’s surface area and thickness are associated with parental internalizing behavior. See supplemental Table S2 for more analysis results on the association between parental behavior problems and children’s limbic brain structures. 

**Figure 1:**
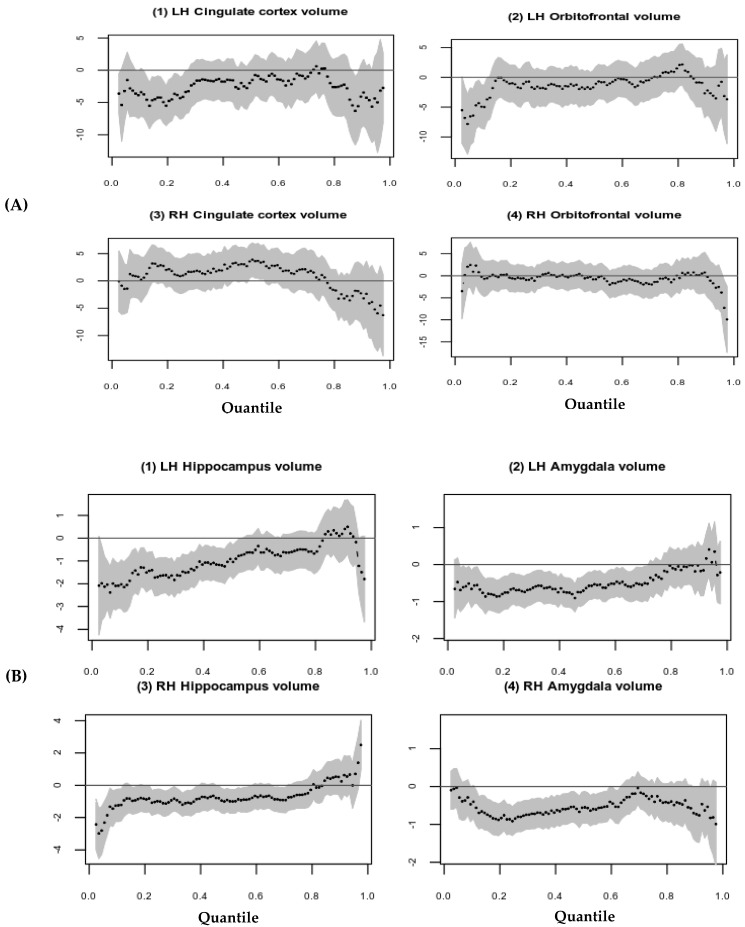
Association between parental internalizing behavior and limbic structures volume (mm^3^) using Quantile regression plots. (**A**) Association between parental internalizing behavior (*y*-axis) and cortical limbic structures (*x*-axis); (**B**) Association between parental internalizing behavior (*y*-axis) and subcortical limbic structures (*x*-axis).

Quantile regression plots visually demonstrated that, at lower quantiles, there are notable associations between parental internalizing behavior and left cortical brain volume structures (see Figure 1A). Parental internalizing behavior may be nonlinearly associated with RH cingulate cortical volume, Figure 1A (3). The parental internalizing behavior is associated with hippocampi and amygdala, except at the higher quantile regressions (Figure 1B), with the strongest parameter estimates reached at approximately the 20th percentile. 

**Figure 2:**
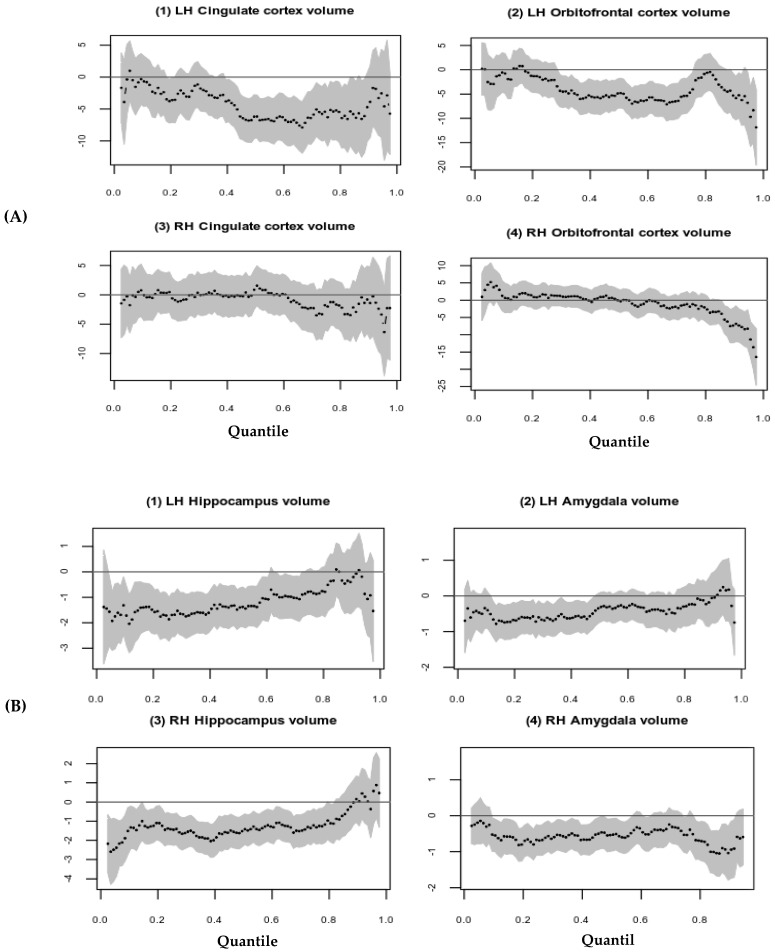
Association between parental externalizing behavior and limbic structures volume (mm^3^) using Quantile regression plots; (**A**) Association between parental externalizing behavior (*y*-axis) and cortical limbic structures (*x*-axis); (**B**) Association between parental externalizing behavior (*y*-axis) and subcortical limbic structures (*x*-axis).

In Figure 2A, quantile regression plots (around 40th to 90th) are showing possible inverse associations between higher parental externalizing behavior and LH cortical brain volume. It is also important to note that there is greater variability in the quantile regression parameter estimates at higher quantiles than at the 40th quantile, where the estimates are more precise with a narrower confidence interval. Parental externalizing behavior is associated with subcortical brain volumes at lower quantiles for both hippocampi and amygdala (see Figure 2B). 

**Figure 3:**
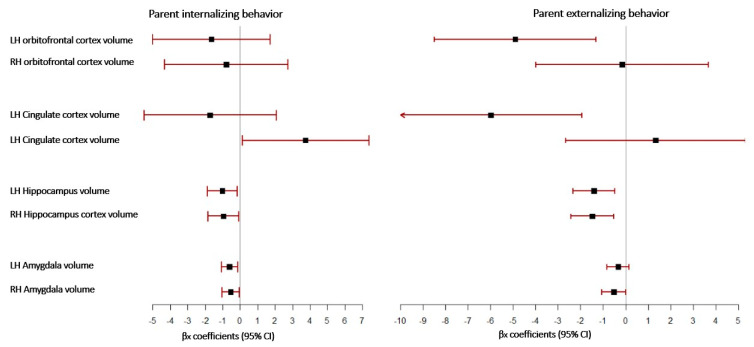
Associations between parental behavior and children limbic brain structures volume (mm^3^) using forest plot.

The forest plot graphically represents the relationship between internalizing and externalizing parental behavior and children’s brain ROI, as illustrated in Figure 3. Each ROI is represented by a line, and the box’s midpoint represents the ROI’s median regression estimate. The width of the line represents the confidence intervals for the median regression estimates for each ROI. Less variance in regression parameter estimates is indicated by a narrower 95% CI, which is more precise. Parental internalizing behavior is associated with the RH cingulate cortex, both hippocampi, and amygdala, while parental externalizing behavior is associated with the LH cingulate cortex, both hippocampi, and RH amygdala.

Finally, we stratified the associations between parental internalizing and externalizing behavior and brain ROI by the children’s gender and cingulate cortex subregions. Higher parental externalizing behavior is associated with reduced LH orbitofrontal cortex volume by −6.39 mm^3^, reduced LH isthmus cingulate cortex volume by −3.30 mm^3^, and reduced both hippocampi by −1.57 to −1.33 mm^3^ in the female brain ROI at *p*-value < 0.05 (see more details in Table S4). However, we observe distinct associations between parental behavior and male brain ROI; higher parental internalizing behavior is associated with increased RH rostral anterior cingulate cortex volume by 1.89 mm^3^ (95% CI: [0.12 to 3.67 mm^3^], *p* = 0.037) and reduced RH hippocampus and LH amygdala volume (Table S5), whereas higher parental externalizing behavior is associated with reduced RH hippocampus by −1.5 mm^3^ (95% CI: [−2.93 to −0.16 mm^3^], *p* = 0.029) (see more analysis results in supplemental Table S6).

## 4. Discussion

In this study, we used ABCD Study data to examine the association between parental internalizing and externalizing behaviors and limbic structures in those parents’ children without mental disorders using quantile regression modeling. We found that parental internalizing and externalizing behaviors are uniquely associated with their children’s limbic brain structures after adjustment for biological confounders and parental socioeconomic status. Specifically, increased parental internalizing behavior is associated with a larger volume RH cingulate cortex and smaller volumes of both hippocampi and amygdalae, whereas increased parental externalizing behavior is associated with smaller volumes of LH cingulate cortex and OFC, and smaller volumes of both hippocampi. The directionality in volume for the RH and LH cingulate cortex here is opposing the normal development according to a previous study [32], and the volume reduction in the OFC is accelerated beside the expected normal reduction during adolescence [19].

From our gender-specific stratified analyses we found several interesting associations: (a) increased parental internalizing behavior is associated with a larger volume RH rostral anterior cingulate, and reduced volume of RH hippocampus and LH amygdala in the males, (b) increased parental externalizing behavior is associated with reduced LH rostral anterior cingulate and RH hippocampus in the male, and (c) in the female, increased parental externalizing behavior is associated with a smaller volume of LH OFC, LH rostral anterior cingulate cortex, and reduced both hippocampi. Hence, parental behavior is associated with male and female children’s limbic structures differently.

These findings suggest that normative variability in parental internalizing and externalizing behaviors is associated with unique limbic structural characteristics in children and adolescents, which may alter the underlying cognitive control of emotion processing and behavior regulation in these brain regions, thereby increasing vulnerability to specific mental disorders later in life. 

In previous studies, it was found that larger RH anterior cingulate cortexes had been reported in adolescents with a negative parental environment [33] and with higher depression symptom scores [34], while reduced LH anterior cingulate volume was reported in adolescents with aggressive behavior and higher risk for alcohol behaviors [35,36]. 

Furthermore, larger, error-related negativity in the anterior cingulate cortex was correlated with higher anxiety symptoms in children and adolescents [8,37]. Also, increased cortical thickness (delayed maturation) in the medial orbitofrontal and posterior cingulate cortex was associated with higher symptoms of anxiety in adolescents [38]. Other studies found reduced LH OFC in adolescents with anhedonia and increased risk of substance abuse [39,40], and disturbed OFC functional connectivity with higher symptoms of anxiety and depression in marijuana-using adolescents [41]. 

The analysis additionally demonstrated that parental internalizing and externalizing behaviors were associated with reduced hippocampus and amygdala in both hemispheres. Most of prior studies reported that smaller hippocampi and amygdalae in children and adolescents were associated with physical abuse, neglect, and low socioeconomic status [5,15], and smaller volume of these two brain structures was associated with more behavioral problems [42] and an increased risk of depression in adolescents [43]. Moreover, studies have shown that adolescents with internalized disorders (anxiety and/or depression) have smaller volume of the hippocampus and amygdala [23,44], while supportive maternal care predicts these two brain structures to be larger in adolescents [45,46].

It’s worth noting that the quantile plots revealed that the hippocampus and amygdala at higher quantiles (larger volume) are not associated with parental internalizing or externalizing behavior. This finding corresponds to a study that discovered resilient adults with a history of childhood adversity have a larger hippocampus and white matter volume, as well as increased connectivity between central executive functions and limbic regions [47]. A larger hippocampus and amygdala may enable some children to develop resilience to mental disorders and behavioral dysfunction later in life. Limbic brain structures are critical for resilience development and more effective in coping with daily life stressors, as well as for improving mental health outcomes [48]. 

Thus, our finding provides support to the hypothesis that normative variability in parental internalizing and externalizing behavior has a significant early influence on the limbic structures of children who do not have mental disorders. Accelerated or delayed structural maturation of these limbic structures may account for some of the inadequacies in adolescent behavior, which may be associated with an increased risk of developing a specific mood disorder or substance abuse later in life. 

To our knowledge, this is the first study that examined the associations between subclinical internalizing and externalizing parental behavior and children’s limbic structures using the quantile (median) regression model. We employed quantile median regression with a robust standard error method, a nonparametric approach, to robustly estimate the association between exposure (parental internalizing and externalizing behaviors) and outcomes (children’s limbic brain structures). Notably, when we visually investigated the effect of parental behavior on the entire spectrum of brain volume by quantile regression plots, it allowed us to narrow down which group of children are more sensitive to their parent’s behavior variability. Therefore, our study findings should be widely applicable, because the ABCD Study population is representative of the general population, and the findings should be reproducible, as it is derived from a robust statistical approach.

Even though we accomplished our study goal using one of the largest, most well-validated cohorts, there are some limitations that may arise from the study design, clinical assessment, and image processing method. We used baseline ABCD data (release 3.0) to examine the association between parental internalizing and externalizing behavior and brain ROI at one time point. Using baseline data which is cross-sectional in nature, we can establish an association between parental behavior and various brain volume markers; however, the association lacks a causal relationship. For establishing a causal relationship, we will need a longitudinal study design that will be able to assess the causal relationship. From a clinical assessment perspective, the second limitation is that the ABCD Study’s baseline data are entirely composed of parent self-reported behavior. As previous research shows the value of multiple informant assessments for an adult is becoming more apparent [49], so it is essential to have an accurate reflection of adult behavior that is not entirely self-reported. 

Another limitation is that the ABCD data 3.0 version analyzed cortical surface-based parcellations and subcortical segmentations using standard ABCD pipelines [28]. Future research could investigate voxel-wise (or volumetric) analytical approaches that may be more appropriate for examining regional differences in the presence of parental internalizing and externalizing behaviors. The ABCD Study used FreeSurfer version 5.3 for automated subcortical segmentation, which has been shown to have some biases in terms of hippocampus and amygdala volume estimation when compared to the manual segmentation method [50]. Future studies may be able to overcome these limitations, as the imaging sciences are continuously evolving with improved software. Lastly, we did not examine the relationship between parental behavior and total brain volume. As this study focuses on the limbic structures of children as an outcome and not the brain as a whole, future studies can be undertaken on the whole-brain analysis. 

## 5. Conclusions

In conclusion, our findings support the hypothesis that parents’ internalizing and externalizing behavior has a significant early impact on the limbic structures of their children who do not have mental disorders or behavior dysfunction, even when biological confounders and parental socioeconomic status are controlled for. Nonetheless, the imbalance in the development of limbic structures reflects the imbalance in the intuitive process for emotion regulation that is associated with increasing vulnerability to adolescent psychopathologies [51]. However, the adaptability of the child and adolescent brain may allow for early intervention prior to onset of symptoms and their progression to clinical levels. Limbic structure MRI markers can be used to identify children at high risk of developing certain mood disorders or substance abuse, allowing for more effective family intervention and a reduction in social burden.

## Figures and Tables

**Table 1:** Baseline descriptive characteristics of ABCD Study population *.

Variables	Mean (SD)	Median [Q1, Q3]
Sample size (n)	11,878	
Age (Years)	9.91 (0.625)	9.92 [9.33, 10.50]
Female (%)	5682 (47.80)	
BMI	19.30 (36.61)	17.65 [15.94, 20.66]
Race (%)		
White	7525 (64.30)	
African American	1869 (16.00)	
Others	2313 (19.80)	
Hispanic (Yes, %)	2292 (19.80)	
Marital status (married/partner, %)	8569 (73.70)	
Parent education (%)		
<HS and HS Diploma	1956 (16.70)	
Some college	3440 (29.40)	
Bachelor’s degree	3316 (28.40)	
Postgraduate degree	2979 (25.50)	
Income (%)		
<$50k	3131 (29.20)	
$50k to <$100k	3046 (28.40)	
$100k to <$200k	3299 (30.80)	
>$200k	1249 (11.60)	

* SD: Standard deviation; Q1: the 25th quartile; Q3: the 75th quartile.

**Table 2:** Associations between parental internalizing behavior and children’s limbic brain structures volume (mm^3^) *.

	β	Robust	t	*p*	95% CI	
		SE			LL	UL
LH Orbitofrontal cortex volume	−1.643	1.722	−0.950	0.340	−5.020	1.733
RH Orbitofrontal cortex volume	−0.779	1.801	−0.430	0.665	−4.309	2.752
LH Cingulate cortex volume	−1.707	1.928	−0.890	0.376	−5.486	2.073
RH Cingulate cortex volume	3.751	1.847	2.030	0.042	0.130	7.373
LH Hippocampus volume	−1.008	0.439	−2.290	0.022	−1.869	−0.147
RH Hippocampus volume	−0.953	0.447	−2.130	0.033	−1.828	−0.078
LH Amygdala volume	−0.599	0.234	−2.560	0.010	−1.058	−0.141
RH Amygdala volume	−0.536	0.257	−2.080	0.037	−1.040	−0.032

* The model adjusted for age, gender, nonwhite race, Hispanic, parental marital status, parental education, and total income. SE: Standard error, t: t-value, *p*: *p*-value, LL: lower level, UL: upper level, LH: left hemisphere, RH: right hemisphere.

**Table 3:** Associations between parental externalizing behavior and children’s limbic brain structures volume (mm^3^) *.

	β	Robust	t	*p*	95% CI	
		SE			LL	UL
LH Orbitofrontal cortex volume	−4.899	1.829	−2.68	0.007	−8.484	−1.313
RH Orbitofrontal cortex volume	−0.156	1.955	−0.08	0.936	−3.989	3.676
LH Cingulate cortex volume	−5.989	2.07	−2.89	0.004	−10.046	−1.931
RH Cingulate cortex volume	1.329	2.037	0.65	0.514	−2.663	5.322
LH Hippocampus volume	−1.413	0.473	−2.99	0.003	−2.341	−0.486
RH Hippocampus volume	−1.478	0.486	−3.04	0.002	−2.43	−0.526
LH Amygdala volume	−0.329	0.247	−1.33	0.183	−0.814	0.155
RH Amygdala volume	−0.517	0.274	−1.89	0.059	−1.054	0.021

* The model adjusted for age, gender, nonwhite race, Hispanic, parental marital status, parental education, and total income. SE: Standard error, t: t-value, *p*: *p*-value, LL: lower level, UL: upper level, LH: left hemisphere, RH: right hemisphere.

## Data Availability

The ABCD Study data repository grows and changes over time. A complete listing of participating sites and study investigators is found at https://abcdstudy.org/study-sites/ (accessed on 10 February 2021).

## Supplementary Materials

The following supporting information can be downloaded at: https://www.mdpi.com/article/10.3390/brainsci12101319/s1; Table S1: Baseline descriptive characteristics of parent t-score; Table S2: Relationship between parental behavior and limbic brain structures surface area (mm^2^) and thickness (mm) in children; Table S3: Relationship between parental internalizing behavior and limbic brain structures volume (mm^3^) in female children; Table S4: Relationship between parental externalizing behavior and limbic brain structures volume (mm^3^) in female children; Table S5: Relationship between parental internalizing behavior and limbic brain structures volume (mm^3^) in male children; Table S6: Relationship between parental externalizing behavior and limbic brain structures volume (mm^3^) in male children; Figure S1: Distribution of outcome measurements/variables of various cortical and subcortical regions; Figure S2: Association between parental behavior and left hemisphere limbic brain structures volume (V) in mm^3^, thickness (T) in mm, area (A) in mm^2^ using Spearman correlation; Figure S3: Association between parental behavior and right hemisphere limbic brain structures volume (V) in mm^3^, thickness (T) in mm, area (A) in mm^2^ using Spearman correlation.
