# Supplementary material for: Effects of Parental Internalizing and Externalizing Behavior Problems on Children’s Limbic Brain Structures—An MRI Study
Source: Brain Sci. 2022 Sep 29;12(10):1319. doi: 10.3390/brainsci12101319 (PMC9599765; doi:10.3390/brainsci12101319)

Supplemental Tables

Table S1: Baseline descriptive characteristics of parent t-score[*].

Variables	Mean (SD)	Median (IQR)
Parent Adult self-report (ASR) [a]		
Internalizing behavior	48.12 (10.55)	48.00 [40.00, 55.00]
Externalizing behavior	45.95 (9.62)	46.00 [38.00, 52.00]

Notes: Source of the data is Adolescent Brain Cognitive Development (ABCD) Study; SD: Standard deviation; IQR: Interquartile range. [*] The t-score is a score compared to norms for each gender at ages 6-18, 18-35, and 36-59, based on national probability samples. A normal t-score is 64 or less, a border clinical t-score is between 65 and 69, and a clinical range is 70 or more. [a] The instrument is from Achenbach System of Empirically Based Assessments.

Table S2: Relationship between parental behavior and limbic brain structures Surface Area (mm^2) and Thickness (mm) in children

Parent internalizing behavior						
					95% CI	
**Cortex surface area**	**β**	**Robust SE**	**t**	**p**	**LL**	**UL**
LH Orbitofrontal cortex	−0.40031	0.52050	−0.77000	0.44200	−1.42059	0.61997
RH Orbitofrontal cortex	−0.04656	0.53553	−0.09000	0.93100	−1.09631	1.00318
LH Cingulate cortex	−0.92548	0.59958	−1.54000	0.12300	−2.10077	0.24982
RH Cingulate cortex	−0.21712	0.56976	−0.38000	0.70300	−1.33397	0.89971
**Cortex thickness**						
LH Orbitofrontal cortex	−0.00012	0.00030	−0.39000	0.69700	−0.00072	0.00048
RH Orbitofrontal cortex	0.00054	0.00033	1.62000	0.10500	−0.00011	0.00119
LH Cingulate cortex	0.00026	0.00064	0.40000	0.68600	−0.00099	0.00151
RH Cingulate cortex	0.00047	0.00057	0.82000	0.41000	−0.00065	0.00158
**Parent externalizing behavior**						
					95% CI	
**Cortex surface area**	**β**	**Robust SE**	**t**	**p**	**LL**	**UL**
LH Orbitofrontal cortex	−1.58648	0.56269	−2.82000	0.00500	−2.68946	−0.48351
RH Orbitofrontal cortex	−0.12346	0.57957	−0.21000	0.83100	−1.25953	1.01261
LH Cingulate cortex	−2.30682	0.65467	−3.52000	0.00000	−3.59009	−1.02355
RH Cingulate cortex	−1.05072	0.63211	−1.66000	0.09600	−2.28978	0.18833
**Cortex thickness**						
LH Orbitofrontal cortex	−0.00034	0.00032	−1.03000	0.30100	−0.00097	0.00030
RH Orbitofrontal cortex	0.00056	0.00036	1.53000	0.12600	−0.00016	0.00127
LH Cingulate cortex	−0.00015	0.00069	−0.22000	0.83000	−0.00151	0.00121
RH Cingulate cortex	0.00086	0.00062	1.38000	0.16800	−0.00036	0.00208

*Quantile regression model adjusted for age, nonwhite race, Hispanic, parental marital status, parental education, and total income. SE: Standard error, t: t-value. *P*: p-value, LL: lower level, UL: upper level.

Table S3: Relationship between parental internalizing behavior and limbic brain structures volume (mm^3) in female children*

	β	Robust SE	t	p	95% CI LL	UL
LH Orbitofrontal cortex volume	−1.663	2.555	−0.650	0.515	−6.671	3.346
RH Orbitofrontal cortex volume	−0.808	2.575	−0.310	0.754	−5.857	4.240
LH Rostral anterior cingulate cortex	−0.334	1.045	−0.320	0.750	−2.383	1.716
RH Rostral anterior cingulate cortex	−0.426	0.863	−0.490	0.622	−2.118	1.266
LH Caudal anterior cingulate cortex	0.069	0.995	0.070	0.945	−1.882	2.020
RH Caudal anterior cingulate cortex	−0.456	1.008	−0.450	0.651	−2.433	1.521
LH Posterior cingulate cortex	0.103	0.973	0.110	0.915	−1.805	2.011
RH Posterior cingulate cortex	0.485	1.059	0.460	0.647	−1.591	2.560
LH Isthmus cingulate cortex	−1.505	0.898	−1.680	0.094	−3.266	0.256
RH Isthmus cingulate cortex	−0.083	0.834	−0.100	0.921	−1.718	1.552
LH Hippocampus volume	−1.001	0.626	−1.600	0.110	−2.227	0.226
RH Hippocampus volume	−0.574	0.640	−0.900	0.370	−1.829	0.681
LH Amygdala volume	−0.209	0.329	−0.630	0.526	−0.854	0.437
RH Amygdala volume	−0.364	0.333	−1.090	0.274	−1.016	0.288

*Quantile regression model adjusted for age, nonwhite race, Hispanic, parental marital status, parental education, and total income. SE: Standard error, t: t-value. *P*: p-value, LL: lower level, UL: upper level.

Table S4: Relationship between parental externalizing behavior and limbic brain structures volume (mm^3) in female children*

	β	Robust SE	t	p	95% CI LL	95% CI UL
LH Orbitofrontal cortex volume	−6.398	2.610	−2.450	0.014	−11.514	−1.280
RH Orbitofrontal cortex volume	−2.353	2.831	−0.830	0.406	−7.903	3.197
LH Rostral anterior cingulate cortex	−1.934	1.065	−1.820	0.069	−4.021	0.153
RH Rostral anterior cingulate cortex	0.122	0.959	0.130	0.899	−1.758	2.001
LH Caudal anterior cingulate cortex	−0.560	1.064	−0.530	0.598	−2.646	1.525
RH Caudal anterior cingulate cortex	−1.350	1.055	−1.280	0.201	−3.418	0.719
LH Posterior cingulate cortex	−0.053	1.047	−0.050	0.960	−2.106	2.000
RH Posterior cingulate cortex	−0.576	1.126	−0.510	0.609	−2.782	1.631
LH Isthmus cingulate cortex	−3.308	0.988	−3.350	0.001	−5.245	−1.371
RH Isthmus cingulate cortex	−1.191	0.875	−1.360	0.174	−2.906	0.525
LH Hippocampus volume	−1.571	0.674	−2.330	0.020	−2.892	−0.250
RH Hippocampus volume	−1.337	0.694	−1.930	0.054	−2.697	0.023
LH Amygdala volume	−0.132	0.355	−0.370	0.710	−0.827	0.563
RH Amygdala volume	−0.333	0.359	−0.930	0.354	−1.037	0.371

*Quantile regression model adjusted for age, nonwhite race, Hispanic, parental marital status, parental education, and total income. SE: Standard error, t: t-value. *P*: p-value, LL: lower level, UL: upper level, LH: left hemisphere, RH: right hemisphere.

Table S5: Relationship between parental internalizing behavior and limbic brain structures volume (mm^3) in male children*

	β	Robust SE	t	p	95% CI LL	95% CI UL
LH Orbitofrontal cortex volume	−1.069	2.429	−0.440	0.660	−5.830	3.692
RH Orbitofrontal cortex volume	−0.167	2.546	−0.070	0.948	−5.157	4.824
LH Rostral anterior cingulate cortex	−0.490	0.950	−0.520	0.606	−2.353	1.373
RH Rostral anterior cingulate cortex	1.895	0.908	2.090	0.037	0.116	3.674
LH Caudal anterior cingulate cortex	−0.179	0.931	−0.190	0.848	−2.003	1.646
RH Caudal anterior cingulate cortex	−0.095	0.980	−0.100	0.923	−2.015	1.826
LH Posterior cingulate cortex	−0.366	1.001	−0.370	0.715	−2.328	1.597
RH Posterior cingulate cortex	0.049	1.106	0.040	0.965	−2.119	2.218
LH Isthmus cingulate cortex	1.025	0.894	1.150	0.252	−0.728	2.777
RH Isthmus cingulate cortex	−0.236	0.869	−0.270	0.786	−1.939	1.467
LH Hippocampus volume	−0.581	0.643	−0.900	0.366	−1.840	0.679
RH Hippocampus volume	−1.233	0.617	−2.000	0.046	−2.442	−0.025
LH Amygdala volume	−0.902	0.335	−2.690	0.007	−1.560	−0.245
RH Amygdala volume	−0.596	0.374	−1.590	0.111	−1.329	0.137

*Quantile regression model adjusted for age, nonwhite race, Hispanic, parental marital status, parental education, and total income. SE: Standard error, t: t-value. *P*: p-value, LL: lower level, UL: upper level, LH: left hemisphere, RH: right hemisphere.

Table S6: Relationship between parental externalizing behavior and limbic brain structures volume (mm^3) in male children*

	β	Robust SE	t	p	95% CI LL	UL
LH Orbitofrontal cortex volume	−4.220	2.656	−1.590	0.112	−9.427	0.987
RH Orbitofrontal cortex volume	2.731	2.783	0.980	0.326	−2.725	8.187
LH Rostral anterior cingulate cortex	−1.518	1.007	−1.510	0.132	−3.492	0.456
RH Rostral anterior cingulate cortex	0.314	0.948	0.330	0.740	−1.544	2.172
LH Caudal anterior cingulate cortex	−1.157	1.008	−1.150	0.251	−3.133	0.818
RH Caudal anterior cingulate cortex	0.209	1.079	0.190	0.846	−1.905	2.323
LH Posterior cingulate cortex	−0.777	1.092	−0.710	0.477	−2.917	1.363
RH Posterior cingulate cortex	0.508	1.206	0.420	0.674	−1.857	2.872
LH Isthmus cingulate cortex	0.769	0.968	0.790	0.427	−1.128	2.666
RH Isthmus cingulate cortex	−0.454	0.949	−0.480	0.632	−2.314	1.406
LH Hippocampus volume	−0.953	0.684	−1.390	0.164	−2.293	0.388
RH Hippocampus volume	−1.541	0.707	−2.180	0.029	−2.928	−0.155
LH Amygdala volume	−0.256	0.364	−0.700	0.482	−0.969	0.458
RH Amygdala volume	−0.541	0.398	−1.360	0.174	−1.322	0.240

*Quantile regression model adjusted for age, nonwhite race, Hispanic, parental marital status, parental education, and total income. SE: Standard error, t: t-value. *P*: p-value, LL: lower level, UL: upper level, LH: left hemisphere, RH: right hemisphere.

Supplemental Figures

Figure S1: Distribution of outcome measurements/variables of various cortical and subcortical regions

a. LH Orbitofrontal cortex volume

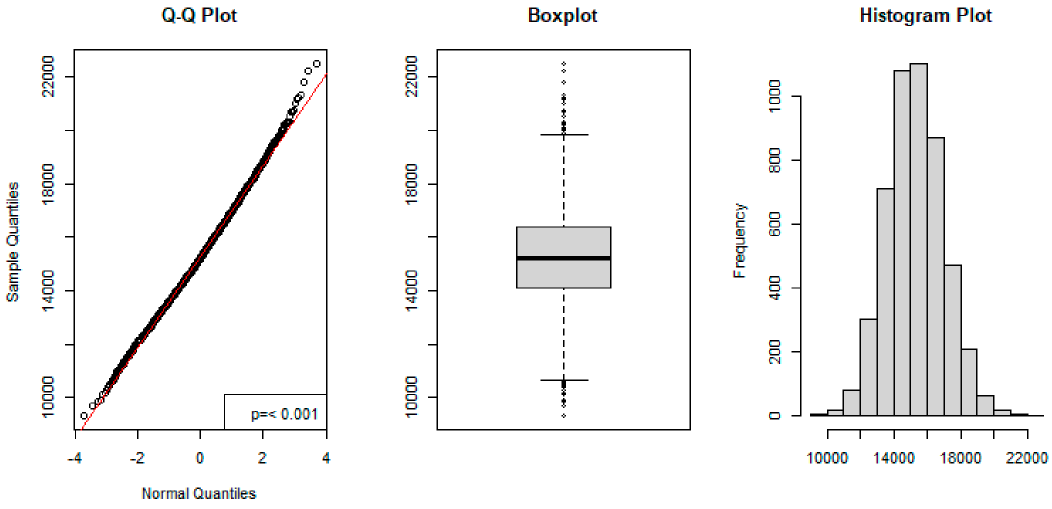

b. RH Orbitofrontal cortex volume

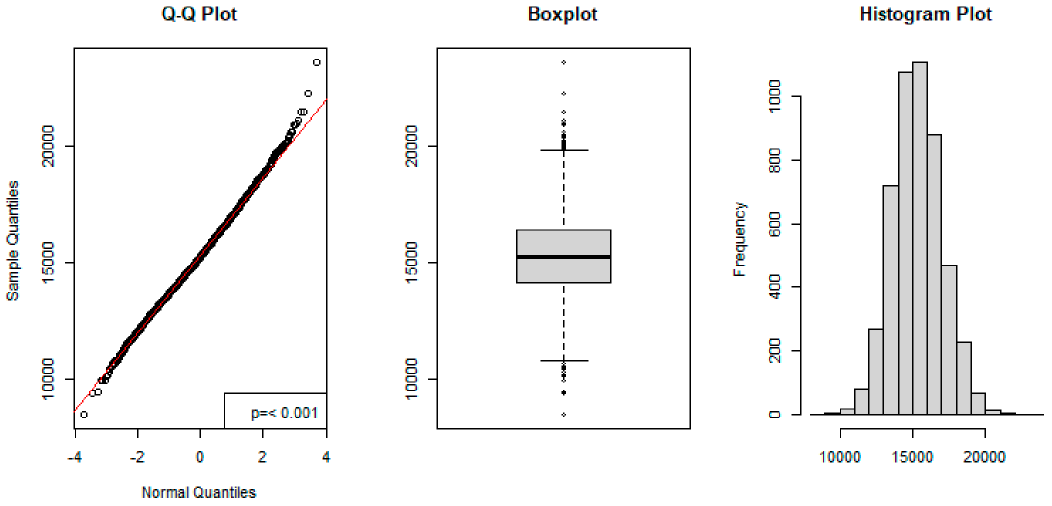

c. LH Cingulate cortex volume

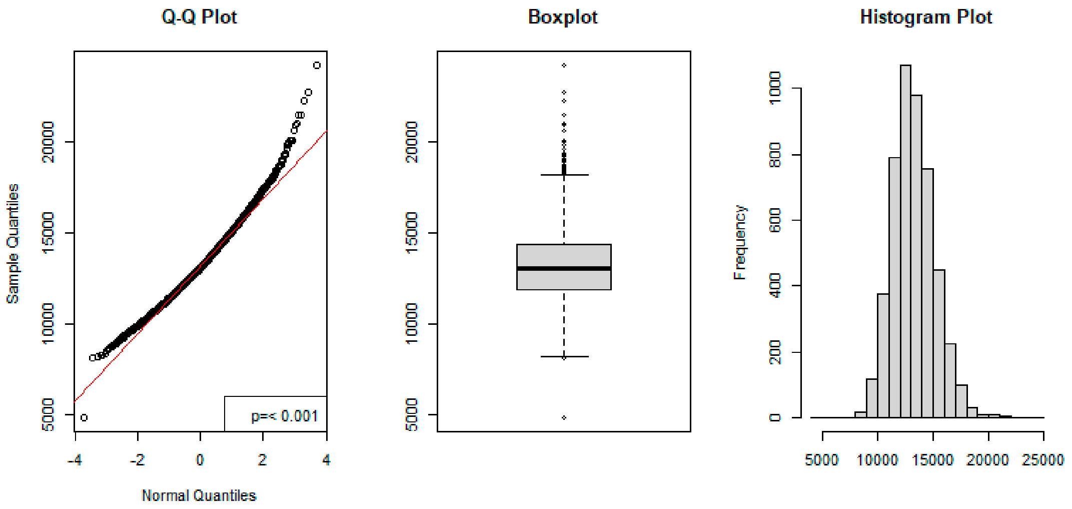

d. RH Cingulate cortex volume

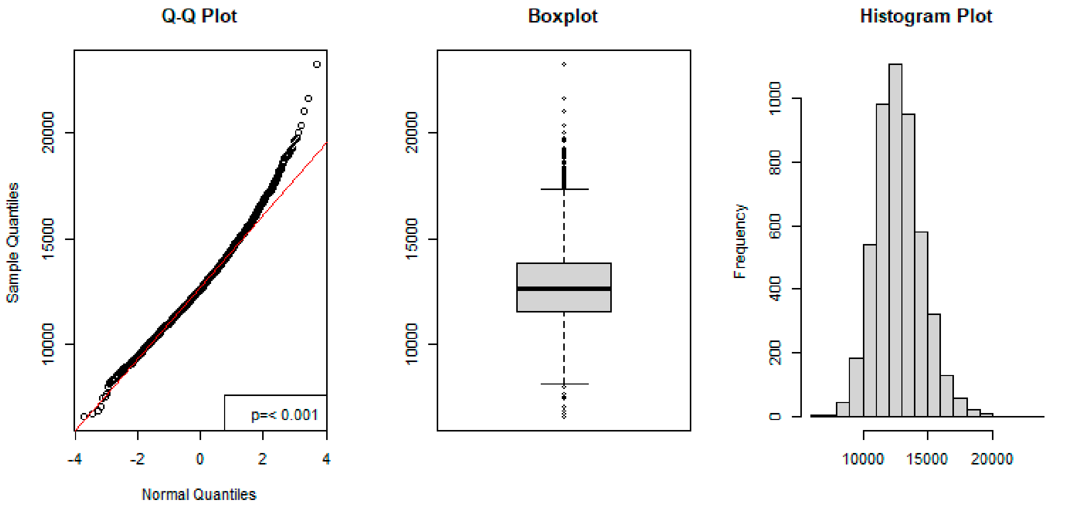

e. LH Hippocampus volume

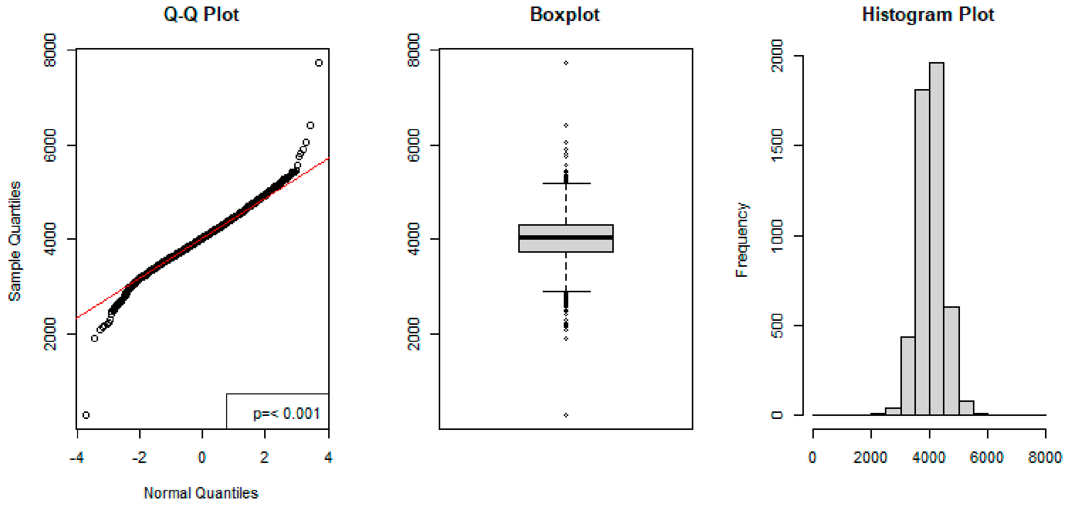

f. RH Hippocampus volume

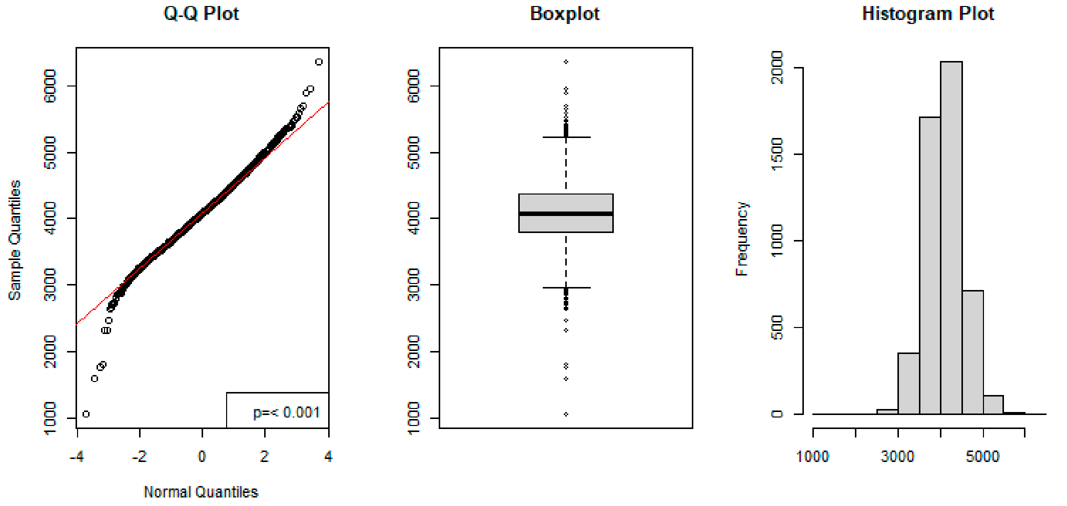

g. LH Amygdala volume

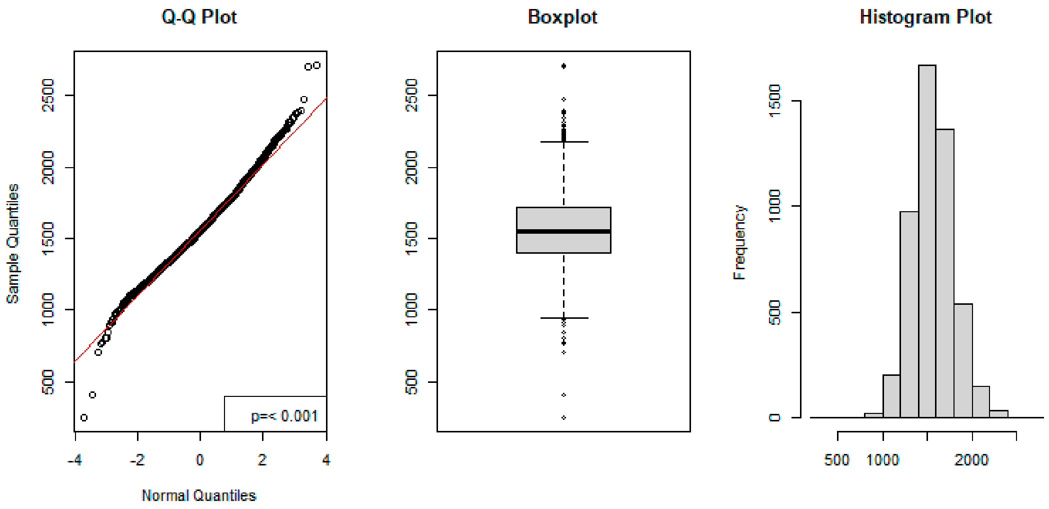

h. RH Amygdala volume

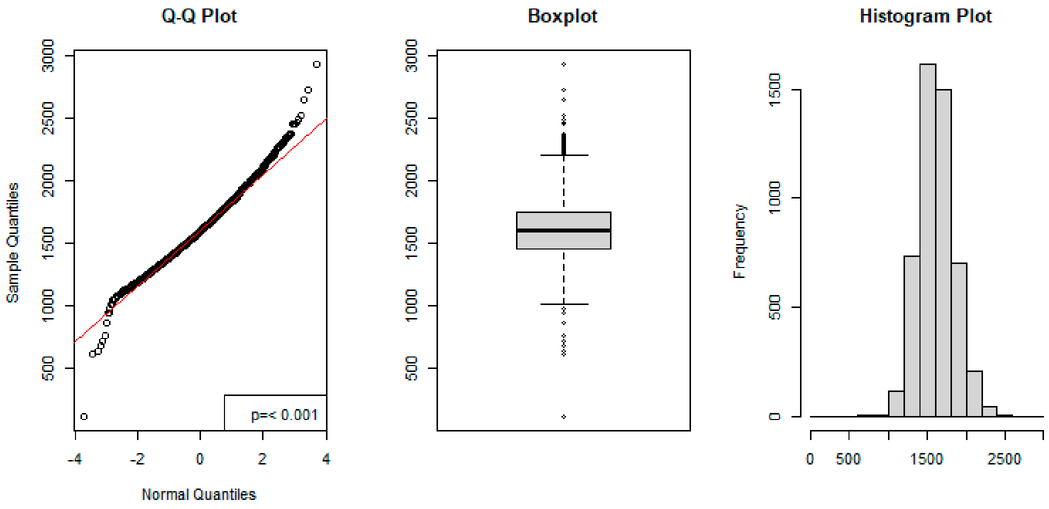

Figure S2: Association between parental behavior and left hemisphere limbic brain structures volume (V) in mm^3, Thickness (T) in mm, Area (A) in mm^2 using Spearman correlation

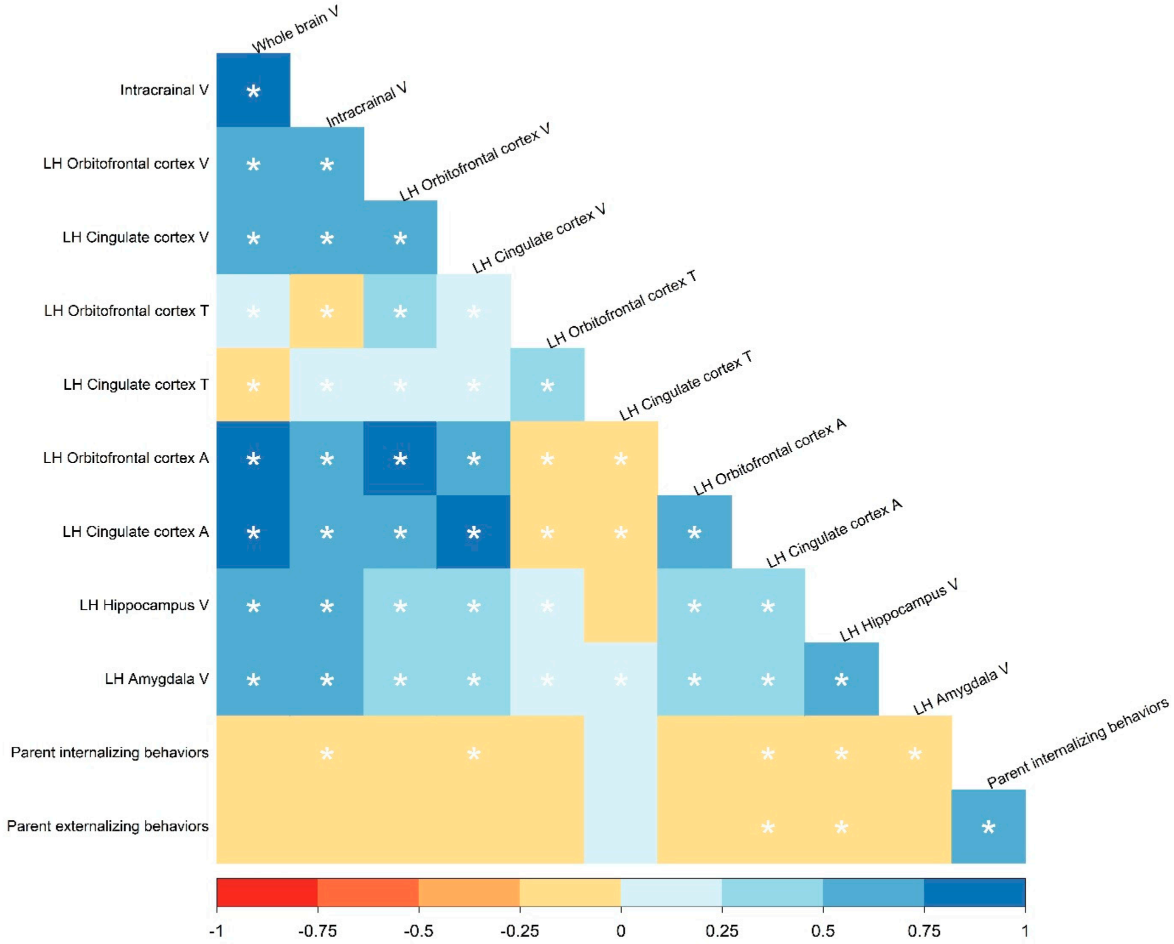

Figure S3: Association between parental behavior and Right hemishpere limbic brain structures volume (V) in mm^3, Thickness (T) in mm, Area (A) in mm^2 using Spearman correlation

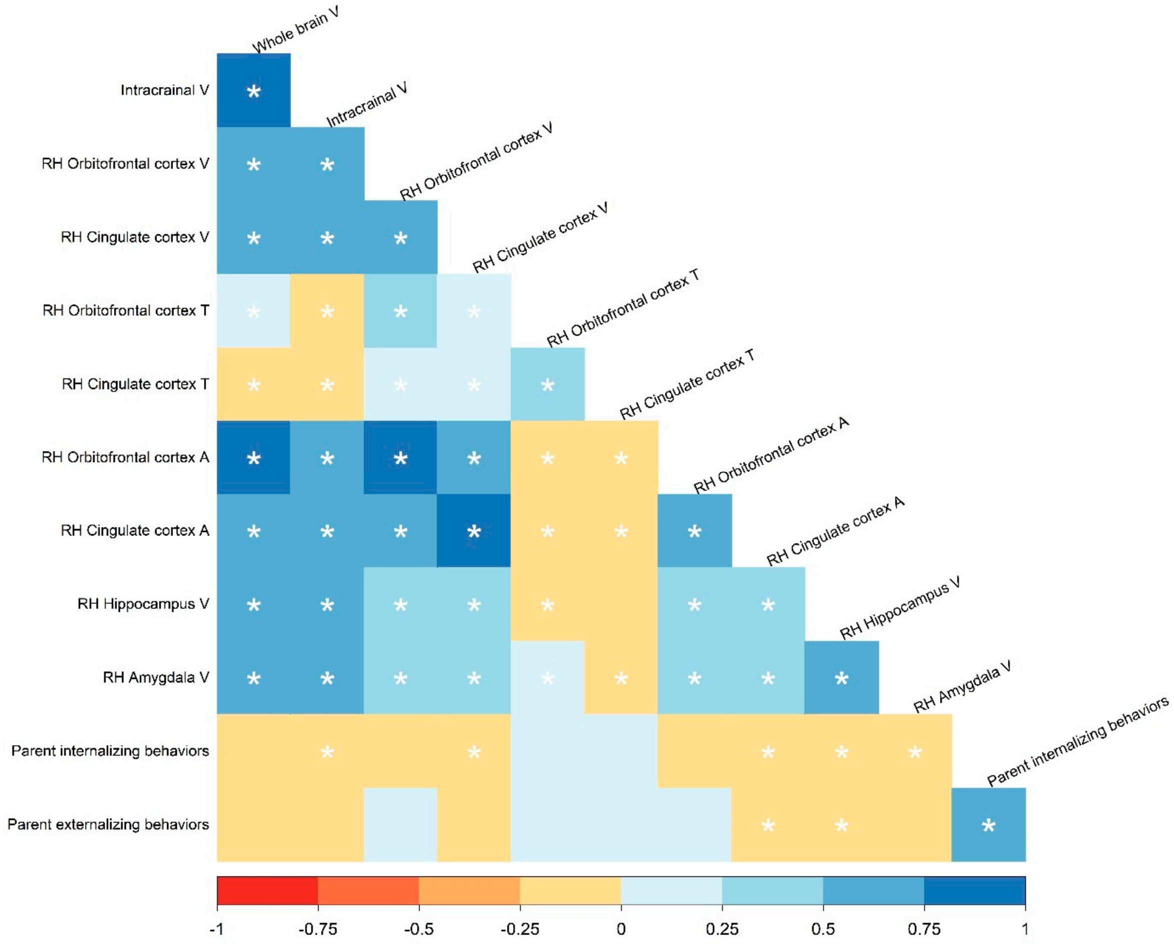

Supplement: Supplementary file 1 [file brainsci-12-01319-s001.zip › brainsci-1869780-supplementary.pdf]
